# Incorporating patient, caregiver, and provider perspectives in the co-design of an app to guide Hospital at Home admission decisions: a qualitative analysis

**DOI:** 10.1093/jamiaopen/ooae079

**Published:** 2024-08-16

**Authors:** Marc Kowalkowski, Tara Eaton, Kelly W Reeves, Justin Kramer, Stephanie Murphy, Colleen Hole, Shih-Hsiung Chou, Amanda Aneralla, Andrew McWilliams

**Affiliations:** Section on Hospital Medicine, Wake Forest University School of Medicine, Winston-Salem, NC 27101, United States; Center for Health System Sciences, Atrium Health, Charlotte, NC 28204, United States; Center for Health System Sciences, Atrium Health, Charlotte, NC 28204, United States; Department of Family Medicine, Atrium Health, Charlotte, NC 28204, United States; Center for Health System Sciences, Atrium Health, Charlotte, NC 28204, United States; Department of Family and Community Medicine, Wake Forest University School of Medicine, Winston-Salem, NC 27104, United States; Medically Home Group, Inc, Boston, MA 02118, United States; Division of Hospital Medicine, Department of Internal Medicine, Atrium Health, Charlotte, NC 28204, United States; Population Health, Clinical Integration, Atrium Health, Charlotte, NC 28204, United States; Medical Group, Atrium Health, Charlotte, NC 28204, United States; Information Technology, Data and Analytics, Atrium Health, Charlotte, NC 28204, United States; ICON plc, Winston-Salem, NC 27103, United States; Division of Hospital Medicine, Department of Internal Medicine, Atrium Health, Charlotte, NC 28204, United States; Information Technology, Medical Informatics, Atrium Health, Charlotte, NC 28204, United States

**Keywords:** Hospital at Home, digital health, decision aid, clinical reasoning, shared decision making

## Abstract

**Objective:**

Hospital at Home (HaH) programs currently lack decision support tools to help efficiently navigate the complex decision-making process surrounding HaH as a care option. We assessed user needs and perspectives to guide early prototyping and co-creation of 4PACS (Partnering Patients and Providers for Personalized Acute Care Selection), a decision support app to help patients make an informed decision when presented with discrete hospitalization options.

**Methods:**

From December 2021 to January 2022, we conducted semi-structured interviews via telephone with patients and caregivers recruited from Atrium Health’s HaH program and physicians and a nurse with experience referring patients to HaH. Interviews were evaluated using thematic analysis. The findings were synthesized to create illustrative user descriptions to aid 4PACS development.

**Results:**

In total, 12 stakeholders participated (3 patients, 2 caregivers, 7 providers [physicians/nurse]). We identified 4 primary themes: attitudes about HaH; 4PACS app content and information needs; barriers to 4PACS implementation; and facilitators to 4PACS implementation. We characterized 3 user descriptions (one per stakeholder group) to support 4PACS design decisions. User needs included patient selection criteria, clear program details, and descriptions of HaH components to inform care expectations. Implementation barriers included conflict between app recommendations and clinical judgement, inability to adequately represent patient-risk profile, and provider burden. Implementation facilitators included ease of use, auto-populating features, and appropriate health literacy.

**Conclusions:**

The findings indicate important information gaps and user needs to help inform 4PACS design and barriers and facilitators to implementing 4PACS in the decision-making process of choosing between hospital-level care options.

## Introduction

Hospital at Home (HaH) was first described in the 1990s as an alternative pathway to costly traditional hospitalization—where care teams deliver acute hospital-level care in a patient’s home.^1^^,^^2^ Over the past 3 decades, an emerging evidence base has demonstrated the safety and efficacy of HaH, either as an admission-avoidance or early supported-discharge substitute for traditional hospitalization.^3–11^ Yet, it was not until the onset of the COVID-19 pandemic in early 2020, and the unprecedented health system pressures that accompanied this global public health emergency, that the HaH care model expanded in scope—with many health systems deploying HaH programs to address surges in demand for hospital care.^12–15^

While the COVID-19 pandemic offered conditions conducive to the rapid implementation of HaH programs (eg, favorable emergency policies, identified critical need, and a well-defined patient population), many experts have questioned whether current expansion can be sustained as urgency surrounding the COVID-19 pandemic subsides. One persistent threat to HaH sustainability is low participation rates. When considering HaH admission, providers and patients must navigate decision-making complexity within a narrow time window. Furthermore, this decision making typically occurs during a time of stress for patients (ie, hospital admission) in a fast-paced, high-acuity clinical environment. Prior research suggests that difficult decisions like this should: (1) incorporate clinical decision support (CDS) informed by validated models to help identify populations at low risk for poor outcomes and (2) be guided by a framework supporting shared decision making (SDM) between providers and patients.^16–18^ Currently, HaH models lack these essential components to guide decision-making.^19^^,^^20^

Creating a widely accepted CDS system is challenged by the inherent complexities in meeting the unique needs of its different user groups (eg, expectations, information quality, format and features, and cultural considerations).^21^^,^^22^ Prior user-centered design applications to address CDS development challenges have successfully leveraged personas (ie, descriptive user models representative of different segments of potential users and their characteristics, perceptions, and behaviors) to enhance user understanding and support design decisions.^23^^,^^24^ Here, we describe the initial step in our user-centered design process to develop a technology-enabled solution, named 4PACS (Partnering Patients and Providers for Personalized Acute Care Selection), to address the gap in CDS tools for HaH. We initially focused on patients with pneumonia given its high prevalence and the availability of validated condition-specific decision support tools tailored to acute hospital settings.^25^^,^^26^ The goal of this qualitative evaluation was to explore patient, caregiver, and provider perspectives and needs for shared HaH decision making to guide early prototyping and co-creation of the 4PACS decision support app to help inform consideration of discrete hospitalization options. In subsequent phases of our work, we will conduct participatory design sessions, usability testing to iteratively refine app development, and a feasibility pilot of the final app version.

## Methods

### Study design

We conducted semi-structured interviews (*N* = 12) with patients and caregivers recruited from our HaH program and physicians and a nurse with experience referring patients to the program via the emergency department (ED) and inpatient care settings. We asked participants about their experiences with and attitudes toward the HaH program, leveraging questions informed by implementation science theory and user-centered design principles. This approach integrating complementary theory-driven models is particularly well suited to eliciting the needs of different stakeholders for decision making and identifying important contextual factors to address during the early design phase.^27^^,^^28^ We followed the EQUATOR network’s Standards for Reporting Qualitative Research.^29^

### Setting and population

The study was conducted at Atrium Health, a large integrated health system headquartered in Charlotte, North Carolina. The study sites included 2 Atrium Health hospitals approved to provide inpatient-level care at home under the Center for Medicaid and Medicare Services’ Acute Hospital Care at Home initiative. Between December 2021 and January 2022, we recruited patients who received HaH treatment for pneumonia, caregivers, and providers (physicians and 1 nurse) to ensure diverse representation from different roles involved in the decision to admit patients to HaH. Eligible participants were approached about study participation by their care team during HaH admission. Members of the study team (M.K., A.M., and T.E.) contacted interested individuals by phone to provide study information and complete enrollment. For provider recruitment, we enrolled ED and Hospitalist providers who had previously admitted patients to HaH. Eligible providers were contacted via email by members of the study team (A.M. and A.A.) regarding study participation. All participants received written study information via email. The study was approved by Advarra IRB (#Pro00054146) with a waiver of signed informed consent.

### Data collection

We conducted semi-structured interviews with 12 total participants—patients (*n* = 3), caregivers (*n* = 2), and providers (*n* = 7). We developed 3 separate interview guides tailored to each stakeholder group, with similar question domains and subdomains across groups (see Supplementary Appendix S1). Each of the interview guides contained questions regarding participant characteristics and targeting (1) perspectives about HaH care and (2) perspectives about HaH admission decision support. Questions about HaH care were informed by the Empathy Map model for design thinking to help understand experiences related to the HaH care delivery model, while questions about decision support were informed by the Consolidated Framework for Implementation Research (CFIR) to help understand contextual factors relevant to app design and future implementation (eg, CFIR domains [constructs]: Individuals [Motivation], Implementation Process [Planning]).^30–32^ Prompted by COVID-19 precautions, interviews were conducted via telephone by a PhD-level trained qualitative health services researcher (T.E.) with extensive experience conducting qualitative research for program evaluations and intervention development. All interviews were conducted in English, recorded, and transcribed.

### Qualitative analysis

Two study investigators (T.E. and K.R.) worked together to devise the coding strategy, which leveraged both inductive and deductive approaches to coding the interview data. One investigator (T.E.) led the initial coding and analysis of the data using this combined approach. Inductive coding was completed using the Constant Comparison Method^33^ to identify categories and larger themes that emerged from review of the interview transcripts, again leveraging the Empathy Map and CFIR models to inform our analysis of perceptions of HaH care and decision making, respectively. Deductive coding was performed to identify user characteristics for developing one illustrative user persona example for each stakeholder group. These personas were created in large part as case examples to promote engagement of participants around design decisions during the planned design session subsequent to the scope of work presented here. User characteristics collected varied by stakeholder group (ie, patients and caregivers [educational background, occupation, living situation, requiring or providing assistance with daily activities], providers [years in practice, academic responsibilities, experience with HaH]). After initial analysis, 2 investigators (T.E. and K.R.) reviewed and discussed the identified themes to ensure accurate and consistent representation of the data. Once the coding of data were finalized, one investigator with training in user persona development (K.R.) determined the list of key user characteristics to guide the user persona development strategy. These personas included background information, including occupation, home situation, hobbies, technological skills and experience, goals, and pain points in relation to projected 4PACS use for HaH admission decisions. Finally, our study team used informal, structured group processes (eg, brainstorming) to build consensus in mapping thematic findings related to app content and information needs and prioritized implementation barriers to potential design elements for early prototype development. All qualitative analyses were performed using ATLAS.ti.

## Results

A total of 12 stakeholders participated in qualitative interviews (see Supplementary Appendix S1). Participants included patients (*n* = 3) and caregivers (*n* = 2) with a mean age of 56 and 40 years old, respectively. Approximately 60% were female, 80% white, 60% married, and self-reported overall health was very good (20%), good (40%), or fair (40%). Among clinician stakeholder interviewees (*n* = 7), there were 4 ED and 3 Hospitalist providers. Providers had a mean of 7 years in practice, and all had prior experience referring patients with pneumonia to the HaH program.

### Qualitative results

Using inductive coding methodologies, we identified categories of codes and organized them into 4 emergent primary themes: (1) attitudes about HaH; (2) 4PACS app content and information needs; (3) barriers to 4PACS implementation; and (4) facilitators to 4PACS implementation. Our deductive approach yielded additional findings related to (1) patient demographics (eg, educational background, occupation, living situation) and level of independence with daily activities; (2) caregiver demographics and responsibilities; (3) providers’ clinical role and experience with HaH care model; (4) participants’ savviness with technology; and (5) overall likelihood of using a HaH app. In-depth analyses revealed details described below within the context of user persona development and other pre-specified evaluation domains.

### Attitudes about hospital at home

Interview responses revealed important themes about HaH care expressed by different stakeholder groups (Table 1). Providers perceived value in the opportunity to deliver hospital-level care in a patient’s home. Specifically, they reported excitement about the patient-centeredness of HaH care, prior positive efficacy data related to patient outcomes, and potential to promote more continuity via HaH care. Patient selection for HaH was a primary concern expressed by providers, including difficulties with successfully identifying clinically and socially appropriate candidates and staying up to date on fluctuating admission criteria. Additionally, providers indicated they felt inadequately prepared to comprehensively educate patients on the details of receiving care in HaH. Many also described frustrations about navigating HaH capacity constraints, restricted admission hours, and limited catchment area when considering HaH admission for their patients.

**Table 1.** Illustrative examples of attitudes, content, and information needs by domain and subdomain.

Theme	Category	Illustrative quotations
**Attitudes about HaH**	(+) Promotes patient-centered care	*Patient: “[The care team] came in twice a day took care of everything they needed to take care of. And if they told us what time they would be here, that’s what time they were here. The doctors were always available when they were here so that [we] could talk to them over the phone. And then, yeah, they really, really did a great job taking care of us.”* *Caregiver: “the nursing staff were extremely helpful, available, and it seemed like we could reach them any time of the day that we needed to. And then, of course, the over-the-phone doctor—he just was constantly giving my mom hope and kind of setting her at ease… It was just, like, constant update, constant information, and the people—it just seemed like every interaction, we’re getting good news and, good care.”*
	(+) Possible to achieve improved outcomes	*Provider: “There is probably an infectious disease risk that would exist in the hospital that wouldn’t exist at home, potentially. So that’s a huge benefit.”* *Provider: “staying in the hospital without all their normal surroundings can get pretty depressing, which has a big impact on health. So to be able to have that care at home and have their usual surroundings around them, I just think it helps to improve health for the patients that can do it.”* *Provider: “for older people, especially, if it could cut down on just the delirium that they can experience in the hospital, I think that’s a really positive thing.”*
	(+) Comfort of home	*Patient: “Getting cared for at home… how just the mood of being at home helps you heal! You’re at home. You’re not stressed out like you are at the hospital. There’s something about it. You’ll get better quicker at home than you are going to in the hospital. I’m a believer in that.”*
	(+) Less disruptive to daily activities	*Provider: “So with Hospital at Home, patients can stay at their home, and other than the 1 or 2 hours for the visit time, they are free to do their routine activities, and stay with their family. And, so this is what excites me. If I were a patient, I would prefer getting my treatment at my own home, rather than being in a hospital at an unknown place, with the food, the room, the stay, the sheets. Like, everything is different, right?”* *Patient: “Not having to stay at the hospital—absolutely! That was amazing. No one wants to go to the hospital, or stay there, or have to visit, so that was good.”* *Caregiver: “[they] get to be with their family, around their own surroundings, get to watch their own TV shows. You know, have their own kind of homecooked meals, versus having to be in the hospital and kind of feel restricted from all those ends.”*
	(−) Complex, changing referral process	*Provider: “Criteria [for Hospital at Home] like catchment area and capacity change frequently…and the ED is a hectic place, so…I don't always consider it as an option.”*
	(−) Reduced control of patient care	*Provider: “I definitely don’t get to see really what happens to the patient after they leave my [care], so I do hope that they don’t get missed, or that they get all the things that they need in order to remain stable.”*
	(−) Fear of what to do in emergency	*Provider: “Say patient A is decompensating, but, the EMS assigned to them, they’re seeing patient B at that time, and that patient B is having some complications. It’s kind of a how quick can we get to patient A, and do they need to call 911 first? And I think scares—ends up kind of scaring families and the patients more, like, “Oh, gosh, I’m at home, but I don’t have anyone here.” Whereas, as in the hospital, someone can be there in like 2 minutes.”*
	(−) Caregiver burden	*Caregiver: “It was very hard because I’m not a nurse. But when you love someone, you just take care of them automatically. But everyone who came through to take care of him was amazing and so helpful while I was going through that.”*
**4PACS app content and information needs**	(−) Unclear expectations for care	*Patient: “I think they could better communicate, when you’re released, what’s going to happen when you get home… because when they made the decision to release me, I was like, “What? Okay, good. I want to go, but is everything set up?” I think patients leaving knowing exactly what to expect would help.”*
	(+) Frequently asked questions	*Patient: “Maybe there’s a question center where you can ask a quick set of questions and maybe get some easy and quick answers. Or even like a frequently asked questions section that addresses some of the concerns that people are feeling. Those things would be I think really cool pieces included in an app.”*
	(+) Differences between care options	*Provider: “I feel like it would be very nice to just [be able to] talk about the risks and the benefits of [their different options], and how they qualify, and it just offers me and the patient more confidence in making the right choice for them.”*
	(+) Care team descriptions	*Patient: “I think my biggest initial concern was the idea that the paramedic coming, but after seeing the people who were coming—you can tell they were very well versed. They understood what they were doing. So I would say, yeah, I was comfortable. Like, I never realized the paramedic could come to the house like that. I just—maybe, in my mind, I was expecting more of a typical, like, nursing staff or something along those lines, but that was just due to my ignorance. But that was probably the only thing that initially worried me.”* *Caregiver: “They didn’t really seem to know the right people to contact… It caused my mom to almost start to break down into tears to the point where I had to tell the nurse, “we need to be in contact with the people that are gonna be supplying her care going forward.” I think, maybe, some better communication or better understanding of who to reach out to…”*
	(−) Financial concerns	*Patient: “…anyway, I got something that said my Medicare program was not going to cover the at-home care. A woman from Hospital at Home…sent an appeal for me. So I would just say that that is something that could be very important to go over before making the decision or maybe even having one of the social workers in the hospital look into your program with you.”*
	(+) Clinical risk score information	*Provider: “If they were hesitant to go home I could show them, okay, well, your risk of a bad outcome is extraordinarily low based on this, your clinical features, and your safety is not just something I think clinically would be reasonable, but, also, the app helps provide an actual risk score.”*
	(−) Additional complex patient features	*Provider: “In theory, it’s great, but really it’s all kind of this clinical picture and talking with your patient, and it’s so much more than checkboxes… I feel like sometimes [an app] is just really textbook, and rarely anything in medicine is textbook because there’s just so much more that goes into those decisions…[it is] so much more than 10 checkbox fields.”*

All patients and caregivers said they were satisfied with the HaH care received and shared they appreciated the opportunity to receive hospital care in the comfort of their own home. Additionally, they stated that care felt more personalized, that it met their needs and expectations, and it was beneficial to have more time near family and caregivers, compared to traditional hospitalization. Many also reported feeling well-supported by the HaH care team during admission. Conversely, patients and caregivers expressed concerns related to the lack of program information available prior to HaH admission and the resulting anxiety associated with that knowledge gap. Furthermore, they voiced uncertainty around how to manage their care at home in the event of disease worsening or healthcare emergencies that could arise during the HaH admission without direct, around-the-clock physical presence of healthcare providers (as experienced during a traditional hospitalization).

### 4PACS app content and information needs

Key themes emerged from stakeholder’s responses regarding the content needs to be addressed during the design of 4PACS. Each stakeholder group cited the importance of having accessible, transparent information to describe program components and communicate what to expect when receiving HaH care, prior to the admission decision (ie, to help alleviate knowledge gaps perceived to induce fear). Patients and caregivers also stated it was important to understand how certain elements of care at home differed from routine inpatient care delivery (eg, remote monitoring devices), roles of the involved care team members (eg, when and which clinical team members would visit the patient’s home or interact virtually), and information on costs of care that enables comparison of different treatment options. Providers also emphasized that it was necessary to represent distinct clinical (eg, potential need for procedure) and other nonclinical risk factors (eg, home environment) associated with patient eligibility and appropriateness for HaH selection.

### Barriers and facilitators to 4PACS implementation

Providers reported several potential barriers to 4PACS implementation (Table 2), including the inability to comprehensively represent the multidimensional patient profile to accurately determine risks and assess safety. Additionally, they highlighted the potential for conflict or inconsistencies between app recommendations and clinical judgement, workflow integration challenges, lack of connectivity within the I, and provider burden with app use in the setting of increased time constraints. Provider recommendations to facilitate 4PACS implementation included trust in the validity of data incorporated into 4PACS, inclusion of auto-populating features to reduce the time required to input readily available information, and demonstrating the value of use within the admission workflow. Patients and caregivers also endorsed that 4PACS should be easy to use (eg, simple interface, multimedia, and interactive choices), tailored to appropriate health and reading literacy levels for diverse patient groups, provide clear guidance in response to frequently asked questions about HaH care, and ensure technical support is available, when needed.

**Table 2.** Illustrative examples of barriers and facilitators by domain and subdomain.

Theme	Category	Illustrative quotations
**Barriers to 4PACS implementation**	(-) Information technology integration	*Provider: “If it was a separate app I have to add to my phone that’s already full of apps and then think to use it every time, I don’t know if I’d be as likely. I could see the helpfulness in it, but I wouldn’t be as likely as if it was just on the computer where I was already in the chart.”*
	(-) Compatibility with workflow	*Provider:* “*there’s so many different things they want us clicking and doing. So [it has to be] something easy in my workflow of the day*” *Provider: “I think it’s kind of easy also to forget that you have an app for things like this when you’re trying to round on patients on the inpatient side, especially when it’s really busy.”*
	(-) Increased provider burden	*Provider: “it’s kind of, like, yet another thing to have to fill out and more forms to do and more buttons to click and, yet, another app to have on my phone. So I think, …it’s easier for me to look over and review it and determine eligibility and not have to fill out another data point, which takes more time.”*
	(-) Conflict with clinical judgement	*Provider: “I think that …clinician judgment has to be first and foremost. I think that’s probably the main reason I wouldn’t use it, if I felt like my judgment trumped any risk score [in the app].”*
	(-) Reduced personal interaction	*Patient: “If I’m face to face with a doctor and there’s an app involved, I would prefer just to stay—keep it more personal with the face to face. I, probably, would choose that over using an app.”* *Provider: “if you were talking about using [the app] at the point of care right in front of the patient, I think that’s also poor perspective to use your phone in front of the patient, too, because—well, obviously, you tell them what you’re doing, but that always kinda makes them think that “What’s going on? Why are you on your phone?”*
	(-) Limited digital literacy	*Patient: “some people are not tech savvy, …but they gotta be able to know what they’re getting into”*
	(-) Limited health literacy	*Patient: “I have access to my records, and I read things, and I don’t even have a clue what it’s about. And then I don’t know—do I really want to know more? So I would say if it’s too technical or too much information, it could be a little bit overwhelming.”*
**Facilitators to 4PACS implementation**	(+) System function for providers	*Provider: “The biggest thing is functionality. Like, if it functions and it makes good recommendations and it’s somehow automatically scheduling or putting patients into a database to organize and structure scheduling, …then I’d say that’d be great.”*
	(+) Streamline processes	*Provider: “Streamlining [decision-making & admission] processes would be really nice with an app, and if there were changes that were updated in the app that would just automatically update… that would be a nice feature.”*
	(+) Auto populating features	*Provider: “If it comes prepopulated in the sense, or if [the EHR] can pull all the data and just throw me a score into that app if I just put the patient’s name in—Yes, that would be really useful.”*
	(+) Trust in the tool’s integrated data	*Provider: “…that’s obviously assuming the app’s been calibrated and is fairly smart on pushing you in the right direction and having an algorithm that responds as it should. But, I mean, as long as everything is functional and makes sound decisions, I would absolutely use that.”* *Provider: “So I just wanted to know the data behind the app or the scores to see if they were studied, and what’s their validity, and well are they accurate, and what’s the possibility of accuracy of that app that I’m looking at, and not just blindly, go with the number.”*
	(+) System ease of use for patients	*Caregiver: “I would say the most important key is to make sure it’s easy to navigate… as long as it’s something that is easy to utilize, I think that would be a big help, especially to those who are a little less familiar with [technology].”*
	(+) Appropriate health and reading literacy	*Patient: “If the app had information that was clear to a non-doctor person that might help give a better understanding of what the situation is and why you might [choose] Hospital at Home…if it was really kind of easy for the nonmedical person to see the benefits.”*
	(+) Access to technical assistance	*Caregiver: “Like, you know, you got a 80, 90-somethin’-year-old person in the hospital by themselves, they might not have a clue what to do, how to use it. And some people, you know, they need somebody with them. So I would say a mediator be there to help them with that.”*

### Example user personas

Synthesizing our interview findings for each stakeholder group, we developed 3 distinct, fictitious example user descriptions for patients, caregivers, and providers (Table 3) to help the development team empathetically understand the end user’s behaviors, needs, goals, and challenges for decision making around choosing HaH for pneumonia care. The investigator team created the persona of Violet, a 60-year-old female patient who works in accounting and has an adult child living at home. During our interviews, patients reported experiences with using technology in different areas of their lives but not specific to making decisions about their health. Patients also expressed goals for making health decisions based on clear and precise information, good communication with healthcare professionals involved in their care, and supported by achieving a high-level of confidence in the care decision made. Patients discussed challenges like the lack of reliable information delivered in a hectic and stressful healthcare environment and financial concerns related to healthcare costs and health insurance coverage.

**Table 3.** User personas developed from data collected during semi-structured interviews.

Persona	Description
**Patient** 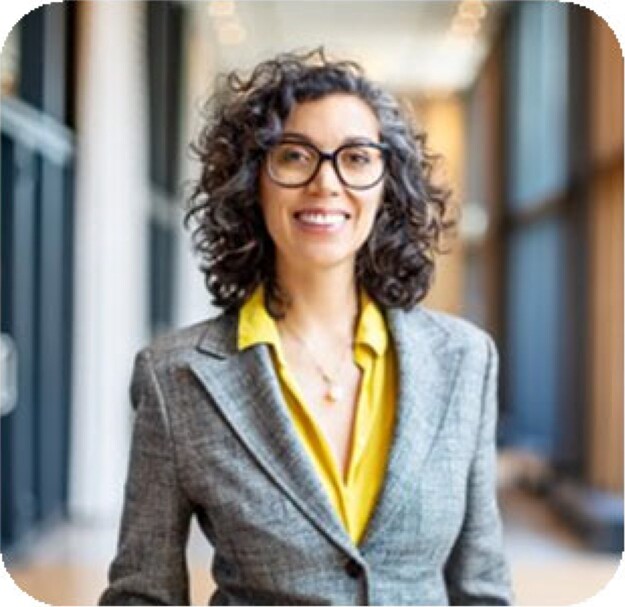 **Violet** *60 years old; works as CPA; adult daughter at home*	Violet is a single female and works as a professional certified personal accountant (CPA). During the COVID-19 pandemic, her adult daughter moved back in with her. When Violet isn’t working, she likes to do yoga to maintain a healthy lifestyle, enjoys wine tasting, and loves to travel. Violet uses technology daily. She relies on technology in many different aspects of her life and is likely to use a health app if it meets her expectations. **Quotes:** “I use technology frequently. I need it to work and not be compromised.” **Goals:** Likes detailed information that is in plain language Wants good communication with her health team Needs to have confidence in the care decision made **Pain points:** Lack of reliable information Concerned about healthcare costs and insurance coverage—is the program covered? What is my out-of-pocket cost?
**Caregiver** 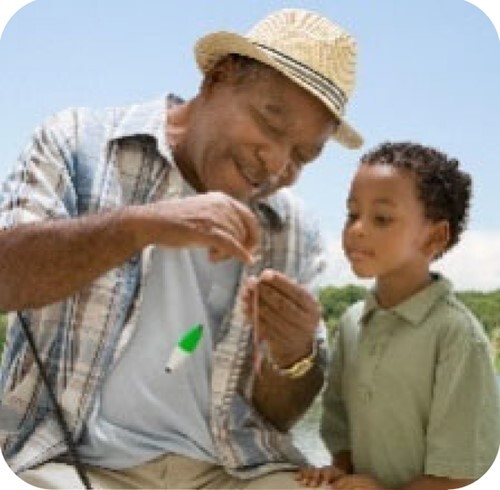 **Joe** *70 years old; retired police officer; lives with wife of 50 years; has 5 adult children who live on their own*	Joe is a married male and is the primary caregiver for his wife of 50 years, who received care in HaH previously. He is a retired police officer and has a network of other retired police friends. As a retiree, he likes to spend his time working in his garden but doesn’t like to cook. Joe also enjoys spending time with his grandchildren. Joe is not very tech savvy and not very comfortable with “new-fangled” technology. He isn’t likely to use a health app, unless it's very straightforward. **Quotes:** “I like things to be simple. If it's complicated, I'm not going to use it.” **Goals:** Wants a good plan with clear instructions and appropriate education Wants to know how to get his questions answered, “Who do I call?” or “Where can I find answers?” Wants to have confidence in the care decision made **Pain points:** Worries about not understanding the plan of care Concerned he won’t know how to reach out for help Anxious he doesn’t have necessary supplies at home to care for his wife
**Provider** 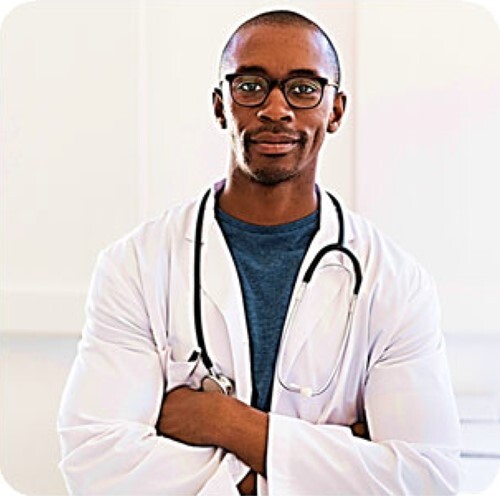 **Dr. Harris** *Emergency Department physician*	Dr. Harris has been an Emergency Department attending physician for 6 years. He lives with his wife and 2 young children. He doesn’t have much free time between work and family responsibilities, but he works out at a gym to manage stress. Dr. Harris is an early adopter of technology. He is likely to use a HaH clinical decision support app if it provides value and is worthwhile. **Quotes:** “There's already so much technology we use. I need the patient's data to be automatically populated and updated so using it would fit in my workflow.” **Goals:** Needs to understand the criteria for any integrated clinical risk stratification tools to have confidence in using an app Wants the process for referral and admission to HaH to be easier Hopes resources can be expanded to extend home-based care offerings into new regions and communities **Pain points:** Worried any app won't provide enough information to accurately determine risk Concerned about increased provider workload Doesn’t want to download “another app”—must be embedded in the EHR system

The investigator team also developed the persona of Joe, a 70-year-old male who functions as the primary caregiver for his wife of 50 years. Joe is retired and has adult children, who do not live at home. During our interviews, caregivers reported wanting simple technology that was easily accessible and provided straightforward information to support decisions about healthcare choices. Caregivers conveyed goals to have well-defined and transparent patient care plans communicated to them by a trusted source and to be included in any education provided to help manage the care needs of their loved ones. Caregivers also highlighted challenges such as not understanding the patient’s care plan, not knowing who to contact with health-related questions and worry about not having the resources to successfully help manage care for their loved ones. The investigators felt these descriptions helped to characterize the uncertainty and anxiety voiced by patients and caregivers when considering decisions about hospital care while coping with the onset of an acute illness.

The last persona created by the investigator team was Dr Harris, a board-certified Emergency Medicine physician with 10 years of experience. Dr Harris is a savvy technology user and is quick to adopt new technology that adds value to his work. During our interviews, providers discussed goals for achieving a clear understanding of the performance and utility of any clinical risk stratification tools being integrated within a decision support application. They also reported wanting easier processes for referral and admission to HaH and greater investment in resources to extend home-based care access to new communities. Challenges described by providers included concerns related to inability of risk stratification tools to accurately determine risk, potential increased workload burden to fit app use into other routine patient care activities, and specific workflow barriers if the app is not effectively integrated within the electronic health record (I) system. The investigators felt these details helped to characterize the strain providers feel when tasked with incorporating new care models and decision-making tools into complex patient care workflows and healthcare environments.

### Mapping to design elements and user interface features

Our in-depth qualitative analysis identified specific needs to be satisfied by design elements integrated into the app prototype (Table 4). To function effectively for intended user groups, we found that design of 4PACS must feature clear, simple language and multimedia to fully describe the program, its components and care teams, and in comparing different care setting options. Additionally, user needs indicate interactive options would be helpful for clarifying relevant care preferences and to track outstanding questions that require discussion with the care team. Finally, there are multiple data requirements related to patient selection that must be addressed by 4PACS to increase trust and utility and reduce burden among users.

**Table 4.** User needs mapped to proposed attribute descriptions and solution requirements.

User need	Design element
HaH program components/description	Clear, simple language and visual aids, written at an 8th grade level (reviewed and approved by health literacy experts)
Alternative means to increase information accessibility	Incorporate video or other media
Understand differences between care settings	Include side-by-side comparisons of key care model differences
Personal values clarification	Check boxes for user selection of preferred care features
Disease risk	Colors, numbers, pictographs to convey disease severity using previously validated risk scores
Social/home environment	Brief, interactive capture of data not readily available in the electronic health record
Access to answers to common questions	Include Frequently Asked Questions section to provide answers in simple, easy to understand format
Question tracking for recall	Simple annotation of outstanding questions for care team
Cost information	Check box/button to request access to hospital financial support staff
Provider burden for data entry	Automated data upload for clinical risk data, other elements

## Discussion

Developing an effective CDS tool to help patients navigate the choice of HaH admission is an important step towards making HaH a sustainable care model. In this qualitative evaluation of stakeholders closest to the decision-making process for admission to HaH, we established that patient eligibility and selection criteria, clear program description to reduce knowledge gaps, and accessible information about the components and team members involved in HaH care represent important user needs to be addressed in early prototype development. Additionally, we found that potential conflict with clinical judgement, inability to comprehensively represent the multidimensional patient-risk profile, and provider burden associated with tool use—particularly in the absence of full I integration—may act as barriers to future implementation of the decision support tool. Conversely, ease of use, auto-populating features, and appropriate health and reading literacy may act as facilitators and should be considered during design. To our knowledge, this is the first study to construct illustrative user personas to inform development of a technology-enabled decision support tool for the SDM surrounding admission to HaH. Eliciting these user descriptions on their perceptions and needs is a significant contribution to the HaH and decision support literature and is particularly important to our own work as a means to promote deep engagement with stakeholders in co-design sessions to guide app development.

Despite slow adoption of the HaH care model at scale, previous research has shown that providers, patients, and caregivers report high levels of satisfaction and positive experiences with home-based hospital care^34^—often attributed to patients’ perceived comfort and familiarity of home during treatment, close proximity and engagement with family and caregivers, facilitation of patient-centered care delivery in the home environment, positive provider-family care dynamics, and evidence showing similar or beneficial health outcomes associated with HaH.^22^ Similarly, in our study, providers’ views of care delivered in HaH were primarily encouraging, with providers expressing enthusiasm for HaH as a care option for their patients. Patients and caregivers also shared positive attitudes similar to those previously described in the literature, and all interviewed participants reported that receiving care in HaH met or exceeded their needs.

Although attitudes of home-based hospital care were noted to be positive by all stakeholder groups engaged in our study, patients and caregivers expressed limited understanding of the HaH model prior to receiving their care at home. Similarly, providers were mixed on perceptions of their ability to effectively educate patients on important aspects of HaH. The lack of information and understanding of HaH services has been shown to deter patients from participating in HaH.^35^ For many common healthcare decisions (eg, health screening, surgery, medication choice), decision aids, often in conjunction with formal or informal SDM processes, have proven helpful to improve patient’s knowledge of the treatment options, accurate risk perceptions, and awareness of what matters most to them.^36^^,^^37^ Additionally, decision aids are likely to support patients making decisions that are aligned with their values and preferences. Our findings reinforce the current gap and need for standardized educational information to be delivered to patients and their caregivers prior to choosing HaH care, specifically related to expectations for care and who, when, what, and how services are delivered.^22^ In our study, participants also shared perceptions about how to effectively address this gap, which align closely to evidence-based recommendations for information sharing in decision support (eg, educational content should use plain language that is accessible to individuals with different levels of health literacy, leverage video components or data visualizations to enhance communication and increase overall understanding, and present choice options that facilitate value clarification important for decision making). These features will be important to help inform our 4PACS app prototype and iterative development process.

Furthermore, making healthcare decisions is inherently complex and involves consideration of multiple health, social, and cultural factors—among providers and patients and their caregivers. The 3 personas developed in our study reflect these influences and present important insights into the motivations and preferences for using a CDS tool in SDM for HaH admission. Of note is the challenge in discussing HaH as a care option without tools to support the efficient identification, screening, and selection of the right patient. Previous studies have found that patient selection is one of the key barriers to HaH participation.^19^ CDS tools have been used successfully to help synthesize relevant data and evidence-based information to enhance care efficiency (eg, pneumonia severity index or CURB-65 scores for informing pneumonia care decisions).^38^ Unique to HaH, clinical risk alone only represents a portion of the comprehensive information necessary to adequately inform decision making. A HaH decision support solution must also incorporate nonclinical factors (eg, social support, patient or caregiver preference, resource availability). Given the care setting requirements for safely receiving hospital-based care in one’s home outside the continuous support and resources of an in-hospital team, these factors potentially play a prominent role in HaH admission decisions and require explicit design focus during tool development.^39^

Finally, we identified a number of workflow considerations to be addressed for 4PACS implementation highlighting the persistent challenges that limit utilization of decision support interventions in clinical practice and the rationale for integrating end-users early in the design.^40^ Within the context of our app design, it will be important to ensure provider burden is limited through auto-populating features when possible and appropriate infrastructure (eg, tool embedded into the I) to support sustainable integration into the care process. Inclusion of patient-facing information (eg, frequently asked questions) may also reduce the burden on providers through more efficient use of time in discussion with patients regarding their care preferences.

Our findings should be viewed within the context of our study design and its inherent limitations. First, this was a single-center study conducted among a small cohort identified via convenience sampling over a short recruitment window (ie, 2 months), and all interviews were completed with English-speaking participants. Additionally, interview data were used to help create user personas representative of distinct user types but may not reflect all users within these stakeholder groups. For example, we did not interview any patients who were younger than 49 years of age, which may limit the representativeness of our study findings to middle-aged and older adults—the group that is most at risk for being hospitalized with pneumonia in the United States.^41^ Despite these limitations, the development of these illustrative user personas effectively achieved our goal to construct meaningful case examples to help ground discussion of user groups’ needs and design requirements during planned participatory design sessions. Additional data collection may be considered to more rigorously develop user personas relevant to HaH decision making, as has been described in the literature.^42^^,^^43^ Finally, the focus of the current study was limited to patients with pneumonia; however, many of our key findings were not disease-specific and are likely to translate across conditions. As one example, users wanted a clear HaH program description to reduce knowledge gaps and concerns, which would be broadly applicable across different diagnoses. However, further work is needed to empirically test the assumptions derived from our current findings in populations with other diagnoses commonly treated in HaH (eg, patients with heart failure).

## Conclusions

Our findings characterize distinct user needs relevant to the HaH admission decision-making process and highlight components that are necessary to integrate into a SDM solution to facilitate an informed choice between hospital-level care options. Incorporating these empirically derived components and iteratively designing with end users will increase the likelihood of creating a useful app that fits in clinical workflows and improves efficiencies, while enabling truly informed decisions. As the next steps in our process, we will conduct design sessions and usability testing with our stakeholders to ideate and refine our 4PACS app prototype; subsequently, we will test the finalized tool in the real-world context of HaH admission decision making.

## Supplementary Material

ooae079_Supplementary_Data

## Data Availability

The datasets generated and analyzed during the study are not available due to participant privacy and ethics restrictions, but the codebook and data collection tools may be available from the corresponding author on reasonable request.

## References

[1] Leff B. Defining and disseminating the hospital-at-home model. CMAJ. 2009;180(2):156-157. 19153385 10.1503/cmaj.081891 PMC2621275

[2] Ritchie C , Leff B. Home-based care reimagined: a full-fledged health care delivery ecosystem without walls. Health Aff (Millwood). 2022;41(5):689-695. 35500180 10.1377/hlthaff.2021.01011

[3] Shepperd S , Iliffe S, Doll HA, et al Admission avoidance hospital at home. Cochrane Database Syst Rev. 2016;9(9):CD007491. 27583824 10.1002/14651858.CD007491.pub2 PMC6457791

[4] Goncalves-Bradley DC , Iliffe S, Doll HA, et al Early discharge hospital at home. Cochrane Database Syst Rev. 2017;6(6):CD000356. 28651296 10.1002/14651858.CD000356.pub4 PMC6481686

[5] Leong MQ , Lim CW, Lai YF. Comparison of Hospital-at-Home models: a systematic review of reviews. BMJ Open. 2021;11(1):e043285. 10.1136/bmjopen-2020-043285 PMC7849878 33514582

[6] Leff B , Burton L, Mader SL, et al Hospital at home: feasibility and outcomes of a program to provide hospital-level care at home for acutely ill older patients. Ann Intern Med. 2005;143(11):798-808. 16330791 10.7326/0003-4819-143-11-200512060-00008

[7] Jeppesen E , Brurberg KG, Vist GE, et al Hospital at home for acute exacerbations of chronic obstructive pulmonary disease. Cochrane Database Syst Rev. 2012;(5):CD003573. 22592692 10.1002/14651858.CD003573.pub2 PMC11622732

[8] Qaddoura A , Yazdan-Ashoori P, Kabali C, et al Efficacy of hospital at home in patients with heart failure: a systematic review and meta-analysis. PLoS One. 2015;10(6):e0129282. 26052944 10.1371/journal.pone.0129282 PMC4460137

[9] Mader SL , Medcraft MC, Joseph C, et al Program at home: a Veterans Affairs Healthcare Program to deliver hospital care in the home. J Am Geriatr Soc. 2008;56(12):2317-2322. 19093932 10.1111/j.1532-5415.2008.02006.x

[10] Cryer L , Shannon SB, Van Amsterdam M, Leff B. Costs for 'hospital at home' patients were 19 percent lower, with equal or better outcomes compared to similar inpatients. Health Aff (Millwood). 2012;31(6):1237-1243. 22665835 10.1377/hlthaff.2011.1132

[11] Levine DM , Ouchi K, Blanchfield B, et al Hospital-level care at home for acutely ill adults: a randomized controlled trial. Ann Intern Med. 2020;172(2):77-85. 31842232 10.7326/M19-0600

[12] Sitammagari K , Murphy S, Kowalkowski M, et al Insights from rapid deployment of a "virtual hospital" as standard care during the COVID-19 pandemic. Ann Intern Med. 2021;174(2):192-199. 33175567 10.7326/M20-4076 PMC7711652

[13] Nogues X , Sanchez-Martinez F, Castells X, et al Hospital-at-Home expands hospital capacity during COVID-19 pandemic. J Am Med Dir Assoc. 2021;22(5):939-942. 33639115 10.1016/j.jamda.2021.01.077 PMC7847393

[14] Clarke DV , Newsam J, Olson DP, Adams D, Wolfe AJ, Fleisher LA. Acute hospital care at home: the CMS waiver experience. Catalyst Non-Issue Content. 2021;2(6). 10.1056/CAT.21.0338

[15] Jaklevic MC. Pandemic boosts an old idea-bringing acute care to the patient. JAMA. 2021;325(17):1706-1708. 10.1001/jama.2021.0127 33851969

[16] Sutton RT , Pincock D, Baumgart DC, Sadowski DC, Fedorak RN, Kroeker KI. An overview of clinical decision support systems: benefits, risks, and strategies for success. NPJ Digit Med. 2020;3:17. 32047862 10.1038/s41746-020-0221-y PMC7005290

[17] Fried TR. Shared decision making—finding the sweet spot. N Engl J Med. 2016;374(2):104-106. 26760081 10.1056/NEJMp1510020

[18] Kunneman M , Montori VM, Castaneda-Guarderas A, Hess EP. What is shared decision making? (and what it is not). Acad Emerg Med. 2016;23(12):1320-1324. 27770514 10.1111/acem.13065

[19] Leff B , DeCherrie LV, Montalto M, Levine DM. A research agenda for hospital at home. J Am Geriatr Soc. 2022;70(4):1060-1069. 35211969 10.1111/jgs.17715 PMC9303641

[20] Gorbenko K , Baim‐Lance A, Franzosa E, et al A national qualitative study of hospital‐at‐home implementation under the CMS acute hospital care at home waiver. J Am Geriatr Soc. 2022;71(1):245-258. 36197021 10.1111/jgs.18071

[21] Westerbeek L , Ploegmakers KJ, de Bruijn GJ, et al Barriers and facilitators influencing medication-related CDSS acceptance according to clinicians: a systematic review. Int J Med Inform. 2021;152:104506. 34091146 10.1016/j.ijmedinf.2021.104506

[22] Chua CMS , Ko SQ, Lai YF, Lim YW, Shorey S. Perceptions of Hospital-at-Home among stakeholders: a meta-synthesis. J Gen Intern Med. 2022;37(3):637-650. 34363185 10.1007/s11606-021-07065-0 PMC8344392

[23] Nielsen L. Personas in use. In: Nielsen L, ed. *Personas—User Focused Design*. London: Springer London; 2019:83-115.

[24] Miaskiewicz T , Kozar KA. Personas and user-centered design: How can personas benefit product design processes? Design Studies. 2011;32(5):417-430.

[25] James DC , Aran S, Ahsan RA, et al Severity assessment tools for predicting mortality in hospitalised patients with community-acquired pneumonia: systematic review and meta-analysis. Thorax. 2010;65(10):878. 20729231 10.1136/thx.2009.133280

[26] Jones BE , Ying J, Nevers M, et al Computerized mortality prediction for community-acquired pneumonia at 117 veterans affairs medical centers. Ann Am Thorac Soc. 2021;18(7):1175-1184. 33635750 10.1513/AnnalsATS.202011-1372OC PMC12131311

[27] Yardley L , Morrison L, Bradbury K, Muller I. The person-based approach to intervention development: application to digital health-related behavior change interventions. J Med Internet Res. 2015;17(1):e30. 25639757 10.2196/jmir.4055 PMC4327440

[28] Haines ER , Dopp A, Lyon AR, et al Harmonizing evidence-based practice, implementation context, and implementation strategies with user-centered design: a case example in young adult cancer care. Implement Sci Commun. 2021;2(1):45. 33902748 10.1186/s43058-021-00147-4 PMC8077816

[29] O’Brien BC , Harris IB, Beckman TJ, Reed DA, Cook DA. Standards for reporting qualitative research. Acad Med. 2014;89(9):1245-1251. 24979285 10.1097/ACM.0000000000000388

[30] Ferreira B , Silva W, Oliveira E, Conte T. Designing personas with empathy map. In: *Proceedings of the 27th International Conference on Software Engineering and Knowledge Engineering*; 2015:501-505.

[31] Damschroder LJ , Aron DC, Keith RE, Kirsh SR, Alexander JA, Lowery JC. Fostering implementation of health services research findings into practice: a consolidated framework for advancing implementation science. Implementation Sci. 2009;4:50. 10.1186/1748-5908-4-50 PMC2736161 19664226

[32] Damschroder LJ , Reardon CM, Widerquist MAO, Lowery J. The updated consolidated framework for implementation research based on user feedback. Implement Sci. 2022;17(1):75. 36309746 10.1186/s13012-022-01245-0 PMC9617234

[33] Bradley EH , Curry LA, Devers KJ. Qualitative data analysis for health services research: developing taxonomy, themes, and theory. Health Serv Res. 2007;42(4):1758-1772. 17286625 10.1111/j.1475-6773.2006.00684.x PMC1955280

[34] Leff B , Burton L, Mader S, et al Satisfaction with hospital at home care. J Am Geriatr Soc. 2006;54(9):1355-1363. 16970642 10.1111/j.1532-5415.2006.00855.x

[35] Paulson N , Paulson MP, Maniaci MJ, Rutledge RA, Inselman S, Zawada SJ. Why U.S. patients declined Hospital-at-Home during the COVID-19 public health emergency: an exploratory mixed methods study. J Patient Exp. 2023;10:23743735231189354. 37560532 10.1177/23743735231189354 PMC10408328

[36] Stacey D , Légaré F, Lewis K, Cochrane Consumers and Communication Group, et al Decision aids for people facing health treatment or screening decisions. Cochrane Database Syst Rev. 2017;4(4):CD001431. 10.1002/14651858.CD001431.pub5 PMC6478132 28402085

[37] Alston C , Paget L, Halvorson G, et al *Communicating with Patients on Health Care Evidence*. Discussion Paper. Washington, DC: National Academy of Medicine; 2012.

[38] Berner ES. Clinical Decision Support Systems: Theory and Practice. 3rd ed. Springer; 2016.

[39] Martins DC , Babajide O, Maani N, et al Integrating social determinants in decision-making processes for health: insights from conceptual frameworks—the 3-D commission. J Urban Health. 2021;98(Suppl 1):51-59. 34480328 10.1007/s11524-021-00560-z PMC8415431

[40] Stacey D , Suwalska V, Boland L, Lewis KB, Presseau J, Thomson R. Are patient decision aids used in clinical practice after rigorous evaluation? A survey of trial authors. Med Decis Making. 2019;39(7):805-815. 31423911 10.1177/0272989X19868193

[41] Williams S , Gousen S, DeFrances C. National hospital care survey demonstration projects: pneumonia inpatient hospitalizations and emergency department visits. Natl Health Stat Report. 2018;(116):1-11. 30248014

[42] Holden RJ , Kulanthaivel A, Purkayastha S, Goggins KM, Kripalani S. Know thy eHealth user: development of biopsychosocial personas from a study of older adults with heart failure. Int J Med Inform. 2017;108:158-167. 29132622 10.1016/j.ijmedinf.2017.10.006 PMC5793874

[43] Holden RJ , Daley CN, Mickelson RS, et al Patient decision-making personas: an application of a patient-centered cognitive task analysis (P-CTA). Appl Ergon. 2020;87:103107. 32310109 10.1016/j.apergo.2020.103107

