# Supplementary material for: Incorporating patient, caregiver, and provider perspectives in the co-design of an app to guide Hospital at Home admission decisions: a qualitative analysis
Source: JAMIA Open. 2024 Aug 16;7(3):ooae079. doi: 10.1093/jamiaopen/ooae079 (PMC11328531; doi:10.1093/jamiaopen/ooae079)
Supplement: ooae079_Supplementary_Data [file ooae079_supplementary_data.docx]

**Supplementary File.**

Supplemental content provided for:

Kowalkowski M, Eaton T, Reeves K, et al. Incorporating patient, caregiver, and provider perspectives in the co design of an app to guide Hospital at Home admission decisions: A qualitative analysis

**Supplementary Appendix Table of Contents**

| **Item** | **Page** |
| --- | --- |
| **S1.** Patient Interview Guide | 3 |
| **S2**. Caregiver Interview Guide | 6 |
| **S3**. Provider Interview Guide | 9 |
| **S4.** Characteristics of study participants | 12 |

**S1. Patient Interview Guide**

|  |
| --- |

| **Introduction**  The Atrium Health Hospital at Home program was designed to give hospital care to patients in their homes. We are interested in learning from our patients and their families about their experiences with the Hospital at Home program. Our goal is to design tools to better support patients and their families so they can choose the hospital care option that is best for them. This interview should last around 20 minutes. You do not need to answer any question that you do not want to. We can also take a break, move on, or stop the interview at any point. Do you have any questions before we get started? |
| --- |

|  |
| --- |

**Section 1: Questions to evaluate patient characteristics.** I would like to begin by getting to know a little bit more about you. I will start by asking you some questions about you and your health.

**Q1**. How long have you lived in the Charlotte area?

**Q2**. What is the highest level of education you completed (like high school or college)?

**Q3**. What do you do for a living?

**Q4**. Who lives in your home with you?

**Q5**. Thinking about your overall health, how would you describe that?

**Q6**. Do you see a primary care provider regularly?

**Q7**. Do you get help for some of the things you do every day, like taking medicine, taking a bath, or preparing meals?

**Q7a**. Probe: If so, who helps you?

**Q8**. Do you regularly care for someone else's health issues?

**Q8a**. Probe: If so, who do you provide care for?

|  |
| --- |

**Section 2: Questions to evaluate patient perspectives on Hospital at Home care.** For the second portion of our interview, I would like to ask you a few questions about your experiences with the Atrium Health Hospital at Home program.

**Q9**. In [insert month], you were taken care of in Hospital at Home. What was that experience like for you?

**Q10**. Thinking back to when you first heard Hospital at Home might be an option for you, what were you excited about?

**Q11**. What worried you about getting hospital care at home?

**Q12**. Did getting care in Hospital at Home meet your needs and expectations?

**Q12a**. Probe: Why or why not?

|  |
| --- |

**Section 3: Questions to evaluate patient perspectives on Hospital at Home admission decision support.** Now we’re almost done. In this final group of questions, I would like to finish by asking you a few questions about the process you went through when choosing Hospital at Home and how you and your doctor arrived at that decision.

**Q13**. When you were considering the option to go to Hospital at Home, what did you think about that helped you make your decision to do it?

**Q14**. At that time, was it difficult for you, in any way, to choose Hospital at Home with your doctor?

**Q11a**. If yes, what was difficult about making that choice?

**Q15**. Do you have any recommendations for what could make choosing Hospital at Home easier for patients?

**Q16**. Imagine you were at the ER (Emergency Department) and there was an app* (like on an iPad or tablet computer) that helped you and your doctor choose the best hospital care option. These options would be getting care in the hospital versus getting hospital care at home. How likely would you be to use an app, like that, to guide your decision-making in choosing the best care option for you?

*To provide further explanation of what the app is, say: “A program where you share information about you (e.g., preferences about treatment, values). It is to help you make better healthcare decisions with your doctor.”

**Q17**. What would make you more likely to use an app like that (with your doctor) when choosing if the Hospital at Home program is the best care option for you?

**Q18**. What would keep you from using an app like that with your doctor?

**Q19**. We are at the end of the interview and I have just one question left to ask. Is there anything else you would like to share about your experiences with the Hospital at Home program?

|  |
| --- |

We are also interested in learning from our patients’ families about their experiences with the Hospital at Home program. Would you have a family member who cares for you that may be interested in participating in a telephone interview, like you did today?

As a next step in our project, we are inviting patients, caregivers (e.g., their family member who cares for them) and doctors to come to a session and help design an app that will help others choose the best type of hospital care to receive. This would probably be a 1.5 hr session. Would you be interested, at all, in taking part in this? If so, a member of our team can contact you. --END

**S2. Caregiver Interview Guide**

|  |
| --- |

| **Introduction**  The Atrium Health Hospital at Home program was designed to give hospital care to patients in their homes. We are interested in learning from our patients and their families about their experiences with the Hospital at Home program. Our goal is to design tools to better support patients and their families so they can choose the hospital care option that is best for them. This interview should last around 20 minutes. You do not need to answer any question that you do not want to. We can also take a break, move on, or stop the interview at any point. Do you have any questions before we get started? |
| --- |

|  |
| --- |

**Section 1: Questions to evaluate caregiver characteristics.** I would like to begin by getting to know a little bit more about you. I will start by asking you some questions about you and your health.

**Q1**. How long have you lived in the Charlotte area?

**Q2**. What is the highest level of education you completed (like high school or college)?

**Q3**. What do you do for a living?

**Q4**. Who lives in your home with you?

**Q5**. Thinking about your overall health, how would you describe that?

**Q6**. Do you see a primary care provider regularly?

**Q7**. Do you regularly care for someone else's health issues?

**Q7a**. Probe: If so, who do you provide care for?

|  |
| --- |

**Section 2: Questions to evaluate caregiver perspectives on Hospital at Home care.** For the second portion of our interview, I would like to ask you a few questions about your experiences with the Atrium Health Hospital at Home program.

**Q8**. In [insert month], your loved one was taken care of in Hospital at Home. What was that experience like for you?

**Q9**. Thinking back to when you first heard Hospital at Home might be an option for your loved one, what were you excited about?

**Q10**. What worried you about your loved one getting hospital care at home?

**Q11**. Did Hospital at Home meet your needs and expectations for your loved one’s care?

**Q11a**. Probe: Why or why not?

|  |
| --- |

**Section 3: Questions to evaluate caregiver perspectives on Hospital at Home admission decision support.** Now we’re almost done. In this final group of questions, I would like to finish by asking you a few questions about the process your loved one went through when choosing Hospital at Home and how they and their doctor arrived at that decision.

**Q12**. Were you present with your loved one when they were considering the option to go to Hospital at Home?

**Q12a**. [If yes to Q12] What did you and your loved one think about, at that time, that helped make the decision to do it?

[If no to Q12] Imagine if you had been there. What would you and your loved one think about to help make the decision to choose Hospital at Home? [Skip Q12b & Q12c. Go to Q13]

**Q12b**. [If yes to Q12] Was it difficult for you and your loved one to choose Hospital at Home with their doctor?

**Q12c**. [If yes to Q12b] What was difficult about making that choice?

**Q13**. Do you have any recommendations for what could make choosing Hospital at Home easier for patients and their families?

**Q14**. Imagine you were at the ER (Emergency Department) and there was an app* (like on an iPad or tablet computer) that helped you, your loved one, and their doctor choose the best hospital care option. These options would be getting care in the hospital versus getting hospital care at home. How likely would you and your loved one be to use an app, like that, to guide your decision-making in helping to choose the best care option for them?

*To provide further explanation of what the app is, say: “A program where you share information about your loved one or family member (e.g., preferences about treatment, values). It is to help you and your loved one or family member make better healthcare decisions with their doctor.”

**Q15**. What would make you and your loved one more likely to use an app like that (with their doctor) when choosing if the Hospital at Home program is the best care option for them?

**Q16**. What would keep you and your loved one from using an app like that with their doctor?

**Q17**. We are at the end of the interview and I have just one question left to ask. Is there anything else you would like to share about your experiences with the Hospital at Home program?

|  |
| --- |

As a next step in our project, we are inviting patients, caregivers (e.g., their family member who cares for them) and doctors to come to a session and help design an app that will help others choose the best type of hospital care to receive. This would probably be a 1.5 hr session. Would you be interested, at all, in taking part in this? If so, a member of our team can contact you. --END

**S3. Provider Interview Guide**

|  |
| --- |

| **Introduction**  The Atrium Health Hospital at Home program is designed to provide hospital care to patients in their home. We are interested in learning from our clinicians about their experiences with the Hospital at Home program. Our goal is to design tools to better support clinicians, patients, and their families in selecting the best hospital care option. This interview should last around 20 minutes. If at any point any of the questions make you feel uncomfortable, you do not need to answer the question. We can also take a break, move on, or stop the interview at any point. Do you have any questions before we get started? |
| --- |

|  |
| --- |

**Section 1: Questions to evaluate provider characteristics.**

I would like to begin by getting to know a little bit about you and your role at Atrium.

**Q1**. Could you tell me about your role within Atrium Health, please?

**Q2**. What is your primary practice specialty?

**Q3**. Approximately how many years have you been in practice?

**Q4**. Are you actively involved in academics as part of your role (e.g. teaching, research responsibilities)?

**Q4a**. Probe: What % of your work effort is dedicated to clinical work?

|  |
| --- |

**Section 2: Questions to evaluate provider perspectives on Hospital at Home care.**

In this next group of questions, I’d like to understand more about your experiences with the Atrium Health Hospital at Home program.

**Q5**. On an average day you are working and admitting patients, what percentage of patients do you consider admitting or transferring to Hospital at Home?

**Q6**. Are there barriers that hold you back from considering more patients for Hospital at Home?

**Q7**. Thinking about the most recent time you admitted a patient to Hospital at Home, what were your reasons for doing so?

**Q8**. For your patients, what excites you about Hospital at Home as an option for their care?

**Q9**. Is there anything that worries or concerns you about Hospital at Home, not meeting the needs of your patients?

|  |
| --- |

**Section 3: Questions to evaluate provider perspectives on Hospital at Home admission decision support.**

In this last group of questions, I’d like to better understand how you think about Hospital at Home admission decisions

**Q10**. When assessing the patient in front of you, what factors do you consider in deciding their eligibility for Hospital at Home?

**Q10a**. I’m not a clinical person. Could you explain what clinical factors would go into your decision? (e.g., vital signs, lab values, radiographic findings [for interviewer’s awareness])

**Q10b**. Could you describe some of the social factors that you might also consider (e.g., home instability, resource needs, insurance [for interviewer’s awareness])

**Q11**. When assessing a patient’s eligibility for Hospital at Home, how do you introduce the idea of Hospital at Home to them?

**Q11a**. Do you discuss the patient’s eligibility with them?

**Q11b**. Is there any discussion of risks or benefits associated with Hospital at Home?

**Q12**. My next question is a scenario question. Imagine that you have determined a patient is a good fit for Hospital at Home. Are there any barriers or facilitators that you have experienced in the next steps of actually getting your patient to Hospital at Home?

**Q13**. Now consider another scenario in which you are assessing a patient’s eligibility for Hospital at Home. Imagine there was an app* available that could incorporate eligibility criteria and a calculated risk score representing the likelihood of a poor outcome (e.g., mortality or need for intensive respiratory or hemodynamic support). How likely would you be to use that at the point of care?

*If provider requests further explanation of what the app is, say: “The app would be a tool intended to present targeted knowledge and person-specific information to aid clinical decision making.”

**Q14**. What would motivate you to use a decision app like that for your patients?

**Q15**. What would discourage you from using an app like that for your patients? (e.g., time constraints, lack of staff support/resources [for interviewer’s awareness])

**Q16**. Is there anything else you would like to share about your experiences with the Hospital at Home program?

|  |
| --- |

As a next step in our project, we will invite patients, caregivers, clinicians, and researchers to plan and design a decision app to help patients and clinicians choose the best hospital care option. We estimate this session would last about 1.5 hours. Does that sound like something you would you be interested in being contacted about to participate in? --END

**S4.** **Characteristics of study participants**

|  | **Patient (n=3)** | **Caregiver (n=2)** |
| --- | --- | --- |
| Mean age, years | 56 | 40 |
| Female | 2 (67%) | 1 (50%) |
| Race/ethnicity |  |  |
| Black | 1 (33%) | 0 |
| White | 2 (67%) | 2 (100%) |
| Married | 2 (67%) | 1 (50%) |
| Insurance status |  |  |
| Commercial | 2 (67%) | 2 (100%) |
| Medicare | 1 (33%) | 0 |
| Highest education |  |  |
| High school diploma | 1 (33%) | 1 (50%) |
| More than high school | 2 (67%) | 1 (50%) |
| Employment status |  |  |
| Employed | 2 (67%) | 2 (100%) |
| Unemployed | 0 | 0 |
| Retired | 1 (33%) | 0 |
| Regular primary care provider |  |  |
| Yes | 2 (67%) | 1 (50%) |
| No | 1 (33%) | 1 (50%) |
| Self-reported overall health |  |  |
| Very good health | 0 | 1 (50%) |
| Good Health | 2 (67%) | 0 |
| Fair health | 1 (33%) | 1 (50%) |
|  |  |  |
|  | **ED provider (n=4)** | **Hospitalist (n=3)** |
| Mean years in practice (min, max) | 8 (5-9) | 7 (3,13) |
| Pct. of time spent in patient care |  |  |
| 50% or less | 0 | 0 |
| More than 50% | 4 (100%) | 3 (100%) |
